# Homologous recombination pathway may play a major role in high-LET radiation-induced DNA double-strand break repair

**DOI:** 10.1093/jrr/rrt181

**Published:** 2014-03

**Authors:** Ariungerel Gerelchuluun, Jiayun Zhu, Fengtao Su, Aroumougame Asaithamby, David J. Chen, Koji Tsuboi

**Affiliations:** 1 Proton Medical Research Center, Graduate School of Comprehensive Human Sciences, University of Tsukuba, 1-1-1 Tennodai, Tsukuba, Ibaraki 305-8575, Japan; 2 Department of Radiation Oncology, University of Texas Southwestern Medical Center, Dallas, TX, USA

**Keywords:** high LET particle, NHEJ and HR pathway, chromosome aberration

## Abstract

**Purpose:** Particle beams are increasingly applied to various cancer treatments because of their excellent dose localization to tumors while preserving surrounding normal tissues. However, characteristic of DNA damages induced by particle beams and their repair mechanisms are not fully understood. It is known that the majority of DNA double-strand breaks (DSBs) induced by ionizing radiation are repaired either by non-homologous end-joining (NHEJ) or by homologous recombination (HR) pathways. However, it has not been clarified how NHEJ and HR pathways contribute to the repair of DSBs induced by various particle beams [1, 2]. Thus, the purpose of this study is to clarify how these repair pathways contribute to the DSB repair in cells after irradiation with various radiation qualities.

**Material and methods:** A control Chinese hamster ovary (CHO) cell line (AA8), its mutant cell line deficient of DNA-PKcs (V3), XRCC4 (XR1) and Chinese hamster lung fibroblast cell line deficient of XRCC2 (IRS1) were exposed to gamma rays, protons, carbon ions and iron ions. V3 and XR1 lack NHEJ pathway, while IRS1 lacks HR pathway. After each irradiation, colony survival and gross-chromosome aberration were examined.

**Results:** It was demonstrated that colony survival was clearly dependent on the presence of NHEJ or HR pathways as well as radiation qualities. Although HR-deficient cells (IRS1) became more sensitive as LET value increased, NHEJ-deficient cells (V3 and XR1) did not further sensitized as LET value increased (Fig. 1). In addition, values of relative biological effectiveness of iron beams were higher in HR-deficient cells than in NHEJ-deficient cells (3.2 in AA8; 2.7 in IRS1; 1.8 in XR1 and V3). These may suggest that HR plays an important role in repairing DNA lesions induced by high-LET radiation. As for the incidence of total chromosomal aberration, we found that its incidence increased as LET values increased in wild-type (AA8) and NHEJ-deficient cells (V3, XR1), but not in HR-deficient cells (IRS1) (Table 1). However, occurrence of chromosome-type aberrations increased as LET values increased in all cell lines analysed here. This may indicate that the chromosomal aberrations occur from not only unrepaired damages but also the repair process of error-prone NHEJ pathway, suggesting that limited capacity of NHEJ to repair DSBs induced by high-LET irradiation may cause increased number of chromosome-type aberrations.

**Conclusions:** Taken together, although NHEJ pathway is the major pathway to repair DSBs induced by various types of radiation, HR pathway may play more important roles as LET value increases.

**Fig. 1.**
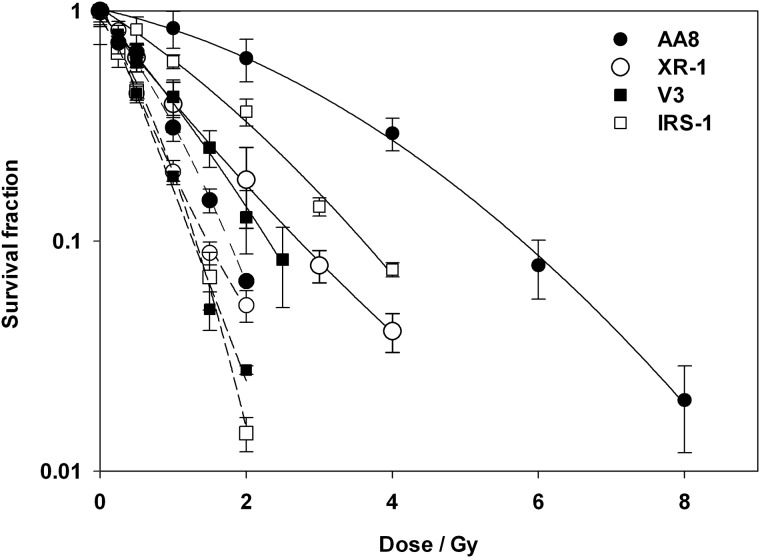
Radio sensitivity after gamma ray and iron beam. Clonogenic survival curves of AA8 (closed circle); XR1 (open circle); V3 (closed square) and IRS1 (open square) after irradiation with gamma ray (dashed) and iron beam (dotted). NHEJ-deficient cells are more sensitive to gamma ray, but HR-deficient cells are most sensitive to iron beam.

**Table 1.** Chromatid and chromosome type aberration per chromosome

Cell lines	Type	Control	Proton	Carbon	Iron
AA8 (wild-type)	Chromatid	0.86	0.24	0.61	1.07
	Chromosome	0.05	1.18	0.74	3.44
XR1 (NHEJ)	Chromatid	0.05	3.18	1.55	3.41
	Chromosome	0	2.59	2.50	4.96
V3 (NHEJ)	Chromatid	0.48	3.37	2.41	7.89
	Chromosome	0.16	5.26	4.97	8.01
IRS1 (HR)	Chromatid	0.02	8.26	10.35	7.59
	Chromosome	0.01	4.24	7.43	8.18

Radio sensitivity after gamma ray and iron beam. Clonogenic survival curves of AA8 (closed circle); XR1 (open circle); V3 (closed square) and IRS1 (open square) after irradiation with gamma ray (dashed) and iron beam (dotted). NHEJ-deficient cells are more sensitive to gamma ray, but HR-deficient cells are most sensitive to iron beam.

Chromatid and chromosome type aberration per chromosome

